# MLKL signaling regulates macrophage polarization in acute pancreatitis through CXCL10

**DOI:** 10.1038/s41419-023-05655-w

**Published:** 2023-02-24

**Authors:** Cheng Peng, Guangping Tu, Jiale Wang, Yilin Wang, Peng Wu, Li Yu, Zhiqiang Li, Xiao Yu

**Affiliations:** 1 Department of Hepatopancreatobiliary Surgery, Third Xiangya Hospital, Central South University, Changsha, 410013 Hunan China; 2 Department of Radiology, Third Xiangya Hospital, Central South University, Changsha, 410013 Hunan China

**Keywords:** Acute inflammation, Innate immunity

## Abstract

Acute pancreatitis (AP) is a disease characterized by local and systemic inflammation with an increasing incidence worldwide. Receptor-interacting serine/threonine protein kinase 3 (RIPK3), mixed-lineage kinase domain-like protein (MLKL), and innate immune cell macrophages have been reported to be involved in the pathogenesis of AP. However, the mechanisms by which RIPK3 and MLKL regulate pancreatic injury, as well as the interactions between injured pancreatic acinar cells and infiltrating macrophages in AP, remain poorly defined. In the present study, experimental pancreatitis was induced in C57BL/6J, *Ripk3*^-/-^ and *Mlkl*^-/-^ mice by cerulein plus lipopolysaccharide in vivo, and primary pancreatic acinar cells were also isolated to uncover cellular mechanisms during cerulein stimulation in vitro. The results showed that MLKL and its phosphorylated protein p-MLKL were upregulated in the pancreas of the mouse AP model and cerulein-treated pancreatic acinar cells, independent of its canonical upstream molecule *Ripk3*, and appeared to function in a cell death-independent manner. Knockout of *Mlkl* attenuated AP in mice by reducing the polarization of pancreatic macrophages toward the M1 phenotype, and this protective effect was partly achieved by reducing the secretion of CXCL10 from pancreatic acinar cells, whereas knockout of *Ripk3* did not. In vitro neutralization of CXCL10 impaired the pro-M1 ability of the conditioned medium of cerulein-treated pancreatic acinar cells, whereas in vivo neutralization of CXCL10 reduced the polarization of pancreatic macrophages toward M1 and the severity of AP in mice. These findings suggested that targeting the MLKL-CXCL10-macrophage axis might be a promising strategy for the treatment of AP.

## Introduction

Acute pancreatitis (AP) is a common gastrointestinal critical disease with an increasing incidence worldwide [1]. Mild acute pancreatitis (MAP) presents with local inflammation, whereas severe acute pancreatitis (SAP) is characterized by systemic inflammatory response syndrome (SIRS), which carries an overall mortality rate of up to 30% [2]. Identification of the pathogenesis of AP is critical for developing specific therapeutics for clinical use.

Necroptosis is a caspase-independent programmed cell death (PCD) with strong proinflammatory properties [3]. Knockout of receptor-interacting serine/threonine protein kinase 3 (RIPK3) or mixed-lineage kinase domain-like protein (MLKL), the key regulators of the necroptotic pathway, can reduce the severity of renal ischemia‒reperfusion (I/R) injury [4, 5] and ischemic stroke [6]. The necroptosis inhibitor necrostatin-1 (Nec-1) has also been reported to alleviate ischemic stroke [7], intestinal I/R-associated liver damage [8], and subarachnoid hemorrhage [9]. However, Nagy et al. found that *Ripk3*^-/-^ mice were not protected from high-fat diet (HFD)-induced liver injury, whereas knockout of *Mlkl* exerted a protective effect [10, 11], which suggested that the molecular machinery in the necroptotic pathway is complex and varies among diseases. In addition, multiple noncanonical mechanisms for RIPK3 and MLKL have been identified [12–20], some of which were even independent of inflammation and necroptosis [15, 17, 18, 20]. The activation of RIPK3 [21–25] and MLKL [25, 26] signaling were also involved in the pathogenesis of AP; however, their contribution to disease severity remains controversial. Therefore, it is necessary to uncover the mechanisms by which RIPK3 and MLKL regulate pancreatic injury in AP.

The phenotype and role of macrophages in inflammation have been well documented [27]. Macrophages are the most abundant immune cells in the inflamed pancreas in the early phase of AP and predominantly present as a proinflammatory M1 phenotype [28, 29]. Thus, some promising intervention modalities attenuate the severity of AP by targeting macrophage polarization imbalance [30–32]. The intracellular contents (which include damage-associated molecular patterns (DAMPs)) released by tissue cells can form a unique proinflammatory microenvironment that in turn influences the polarization status of infiltrating macrophages [3, 27]. For example, Yang reported that ischemia-induced neuronal injury promotes the polarization of macrophages toward the M1 type, while ablating *Ripk3* or *Mlkl* could switch the activation of macrophages from the M1 to the M2 type [6]. However, the interactions between pancreatic acinar cell injury and infiltrating macrophages in AP remain poorly defined.

In the present study, we found that MLKL regulated macrophage polarization in a *Ripk3*-independent manner in mice with AP, and its effect was mediated in part through CXCL10 release. In vitro neutralization of CXCL10 impaired the pro-M1 ability of the conditioned medium of cerulein-treated pancreatic acinar cells, whereas in vivo neutralization of CXCL10 reduced the polarization of pancreatic macrophages toward M1 and the severity of AP in mice.

## Results

### MLKL signaling was activated in acute pancreatitis in a *Ripk3*-independent manner

**Fig. 1:**
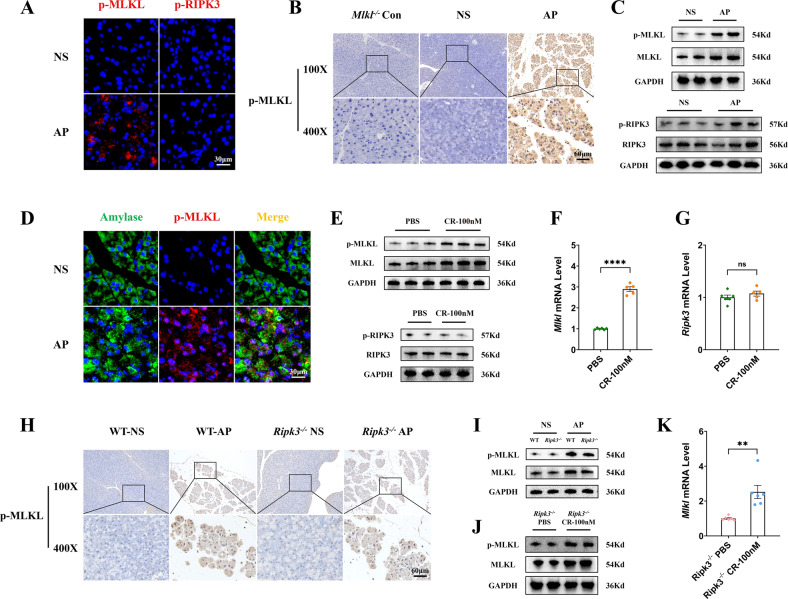
The expression of p-MLKL was upregulated in the pancreas of mice with AP and in cerulein-stimulated pancreatic acinar cells in a *Ripk3*-independent manner. **A** Immunofluorescence staining of p-MLKL (red) and p-RIPK3 (red) in the pancreas of mice (*n* = 3). **B** Immunohistochemical staining of p-MLKL in the pancreas of mice (*n* = 3). **C** Western blot of p-RIPK3, RIPK3, p-MLKL and MLKL in the pancreas of mice. GAPDH was used as the loading control (*n* = 6). **D** Immunofluorescence staining of amylase (green) colocalized with p-MLKL (red) in the pancreas of mice (*n* = 3). **E** Western blot of p-RIPK3, RIPK3, p-MLKL and MLKL in pancreatic acinar cells. GAPDH was used as the loading control (*n* = 7). **F**, **G** mRNA expression of *Mlkl* and *Ripk3* in pancreatic acinar cells, data were normalized to GAPDH (*n* = 5–6). **H** Immunohistochemical staining of p-MLKL in the pancreas of WT and *Ripk3*^-/-^ mice (*n* = 6). **I** Western blot of p-MLKL and MLKL in the pancreas of WT and *Ripk3*^-/-^ mice. GAPDH was used as the loading control (*n* = 6). **J** Western blot of p-MLKL and MLKL in pancreatic acinar cells isolated from *Ripk3*^-/-^ mice. GAPDH was used as the loading control (*n* = 7). **K** mRNA expression of *Mlkl* in pancreatic acinar cells isolated from *Ripk3*^-/-^ mice. Data were normalized to GAPDH (*n* = 6). Values are shown as the means ± SEMs. ns, not significant; *P < 0.05; **P < 0.01; ***P < 0.001; ****P < 0.0001. WT wild type, NS normal saline, AP acute pancreatitis, CR cerulein.

We detected the expression of p-RIPK3 and p-MLKL by immunofluorescence (Fig. 1A and Supplementary Fig. 1A), immunohistochemistry (Fig. 1B and Supplementary Fig. 1B), and western blot (Fig. 1C and Supplementary Fig. 1C, D) in the pancreas from mice with AP induced by cerulein plus LPS [32, 33]. The results showed that the expression of p-MLKL was significantly upregulated relative to MLKL and GAPDH, and interestingly, p-RIPK3 remained low and unchanged. Next, we used amylase in immunofluorescence to label pancreatic acinar cells [34], which account for over 80% of pancreatic parenchymal cells. The results showed substantial colocalization of upregulated p-MLKL and amylase, suggesting that p-MLKL was mainly localized in pancreatic acinar cells (Fig. 1D). Therefore, we extracted primary pancreatic acinar cells from mice and induced injury using cerulein. The results showed a significant upregulation of p-MLKL and *Mlkl* mRNA but not p-RIPK3 and *Ripk3* mRNA (Fig. 1E–G and Supplementary Fig. 1E, F), consistent with the observations from in vivo experiments.

RIPK3 is the upstream molecule of MLKL in the canonical pathway of necroptosis [3]. However, given our finding that in AP, there was a nonparallel change in the levels of p-MLKL and p-RIPK3, we further explored whether the expression of p-MLKL is independent of *Ripk3*. We constructed AP models in WT and *Ripk3*^-/-^ mice, and the results of immunohistochemistry (Fig. 1H and Supplementary Fig. 1G) and western blot (Fig. 1I and Supplementary Fig. 1H) showed that there was a significant upregulation of p-MLKL in the pancreas from both WT mice and *Ripk3*^-/-^ mice with AP. The upregulation of p-MLKL (Fig. 1J and Supplementary Fig. 1I) and *Mlkl* mRNA (Fig. 1K) was also observed in cerulein-induced pancreatic acinar cells extracted from *Ripk3*^-/-^ mice. These results indicated that the expression of p-MLKL was not regulated by *Ripk3* in our models of AP. Since the activation of *Mlkl* was independent of *Ripk3*, what is its possible upstream molecule in this setting? CaMKII can phosphorylate S345 of MLKL in mice to facilitate autophagic flux [15]. In addition, Zhu found that p-CaMKII was persistently highly expressed in the pancreatic tissues of AP mice, and blockade of p-CaMKII using KN93 inhibited the upregulation of p-MLKL and alleviated acinar cell injury both in vivo and in vitro [35]. Consistent with what was observed by Zhu, pancreatic p-CaMKII was found to be significantly upregulated in our study (Supplementary Fig. 2A). Interestingly, there was substantial colocalization of p-CaMKII and p-MLKL (Supplementary Fig. 2B), indicating a possible interaction between them. This result suggested that CaMKII might be the upstream molecule of MLKL, but the specific mechanism still needs further exploration.

### Knockout of *Mlkl*, but not *Ripk3*, attenuated acute pancreatitis in mice

**Fig. 2:**
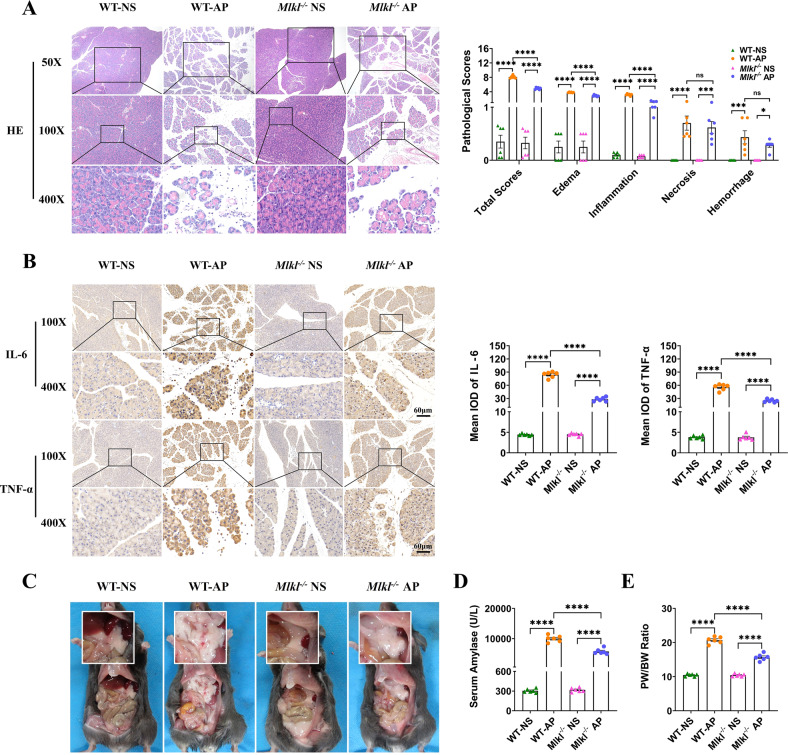
Knockout of *Mlkl* reduced the severity of AP in mice, as indicated by decreased pancreatic pathological scores, expression of inflammatory mediators including IL-6 and TNF-α, extent of pancreatic edema, and level of serum amylase. **A** HE staining and pancreatic pathological scores of the pancreas (*n* = 6). **B** Immunohistochemical staining of TNF-α and IL-6 in the pancreas (*n* = 6). **C** Gross morphology of the pancreas (*n* = 6). **D** Serum amylase (*n* = 6). **E** The ratio of pancreas weight to body weight (*n* = 6). Values are shown as the means ± SEMs. ns, not significant; *P < 0.05; **P < 0.01; ***P < 0.001; ****P < 0.0001. WT wild type, IOD integrated optical density, NS normal saline, AP acute pancreatitis, PW pancreas weight, BW body weight.

We constructed mouse models of AP in WT and *Mlkl*^-/-^ mice. Compared with WT mice with AP, pancreatic pathological scores were significantly lower in *Mlkl*^-/-^ mice with AP, as indicated by a milder degree of edema and less inflammatory cell infiltration in HE staining. Interestingly, the knockout of *Mlkl* did not attenuate the extent of necrosis (Fig. 2A). The levels of pancreatic and serum IL-6 and TNF-α, two key inflammatory mediators in AP [36, 37], were also significantly downregulated in *Mlkl*^-/-^ mice compared with WT mice with AP (Fig. 2B and Supplementary Fig. 3). In addition, the gross morphology of the pancreas (Fig. 2C), serum amylase (Fig. 2D), and the ratio of pancreas weight to body weight (Fig. 2E) also indicated that the knockout of *Mlkl* exerted a protective effect in mice with AP.

**Fig. 3:**
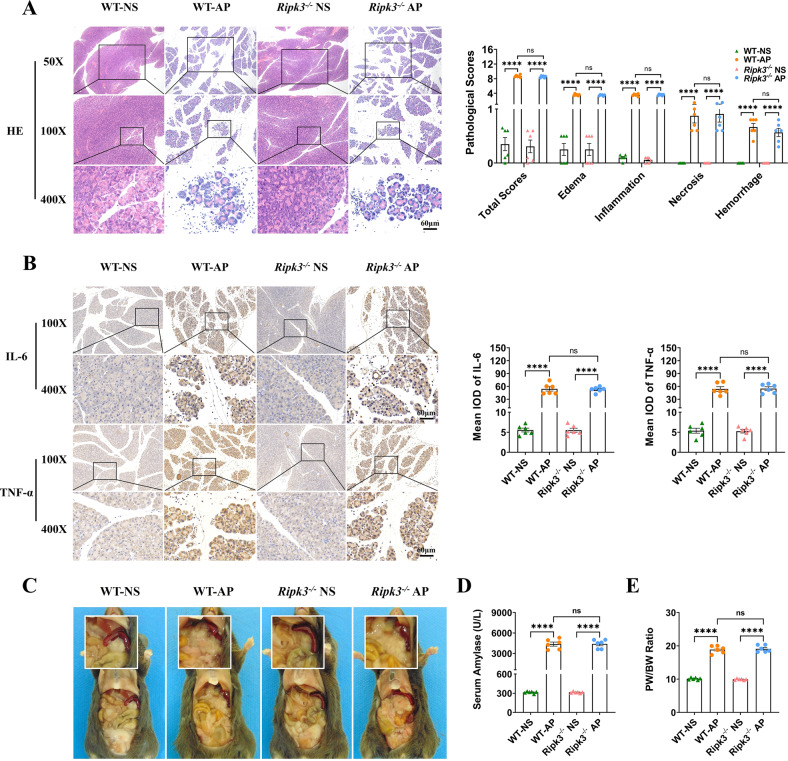
Knockout of *Ripk3* did not exert protective effects against AP in mice. The pancreatic pathological scores, expression of inflammatory mediators including IL-6 and TNF-α, extent of pancreatic edema, and level of serum amylase were not significantly different between WT and *Ripk3*^-/-^ mice with AP. **A** HE staining and pancreatic pathological scores of the pancreas (*n* = 6). **B** Immunohistochemical staining of TNF-α and IL-6 in the pancreas (*n* = 6). **C** Gross morphology of the pancreas (*n* = 6). **D** Serum amylase (*n* = 6). **E** The ratio of pancreas weight to body weight (*n* = 6). Values are shown as the means ± SEMs. ns, not significant; *P < 0.05; **P < 0.01; ***P < 0.001; ****P < 0.0001. WT wild type, IOD integrated optical density, NS normal saline, AP acute pancreatitis, PW pancreas weight, BW body weight.

Given that MLKL and RIPK3 exhibited nonparallel expression levels, which suggested that there may be noncanonical mechanisms in AP, we further explored whether the knockout of *Ripk3* could alleviate AP. Thus, we constructed AP models in WT and *Ripk3*^-/-^ mice. However, there seemed to be no significant difference in pancreatic pathological scores (Fig. 3A), expression of pancreatic and serum IL-6 and TNF-α (Fig. 3B and Supplementary Fig. 3), gross morphology of the pancreas (Fig. 3C), serum amylase (Fig. 3D), and the ratio of pancreas weight to body weight (Fig. 3E) between WT and *Ripk3*^-/-^ mice with AP. The results indicated that knockout of *Ripk3* was not protective in mice with AP.

However, He et al. [23] and Zhang et al. [24] observed that knockout of *Ripk3* alleviated cerulein-induced AP in mice, and Boonchan et al. [25] observed that knockout of *Mlkl* was not protective against cerulein-induced AP in mice, which was inconsistent with our results. In the present study, we constructed a mouse model of AP by administrating cerulein plus LPS, and we questioned whether LPS treatment caused the difference. LPS is an agonist of Toll-like receptor 4 (TLR4) and can activate its downstream NF-κB and MAPK pathways [38, 39]; it can also directly activate caspase11 independently of TLR4 [40]. Therefore, we detected the activation of the NF-κB, MAPK and Caspase11 pathways in WT, *Mlkl*^*-/-*^, and *Ripk3*^*-/-*^ mice with AP and their controls. The results showed that p-NF-kB p65 (p-p65), p-p38 MAPK (p-p38) (Supplementary Fig. 4A) and cleaved caspase11 (Supplementary Fig. 4B) were significantly upregulated in AP mice compared with their controls, but knockout of *Mlkl* or *Ripk3* did not reduce the activation of these pathways, suggesting that LPS did not cause a difference in the severity of AP in mice through the above pathways in the present study.

### Knockout of *Mlkl* alleviated acute pancreatitis in mice by promoting macrophage M2 polarization

**Fig. 4:**
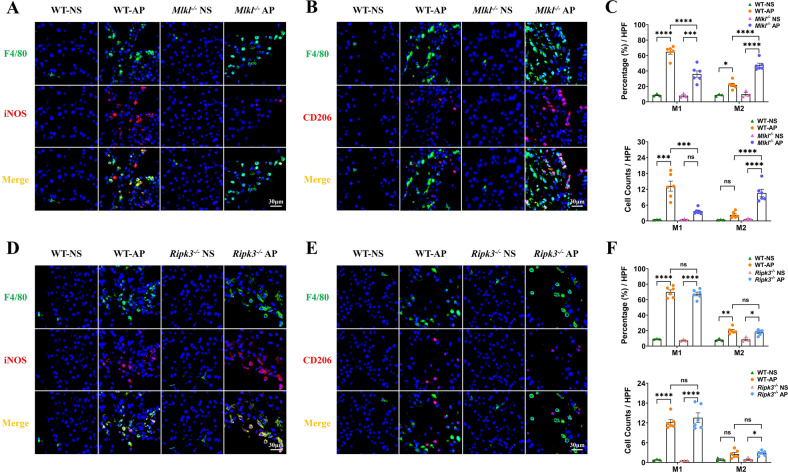
M1 macrophages are enriched in the pancreas of mice with AP. Knockout of *Mlkl* decreased M1 macrophages (F4/80+iNOS+ cells) and increased M2 macrophages (F4/80+CD206+ cells), whereas knockout of *Ripk3* did not change the polarization status of macrophages. **A**, **B** Immunofluorescence staining of F4/80 (green) colocalized with iNOS (red) or CD206 (red) in the pancreas of WT and *Mlkl*^-/-^ mice (*n* = 6). **C** Percentage and cell counts of M1 and M2 macrophages in WT and *Mlkl*^-/-^ mice with AP (*n* = 6). **D**, **E** Immunofluorescence staining of F4/80 (green) colocalized with iNOS (red) or CD206 (red) in the pancreas of WT and *Ripk3*^-/-^ mice (*n* = 6). **F** Percentage and cell counts of M1 and M2 macrophages in WT and *Ripk3*^-/-^ mice with AP (*n* = 6). Values are shown as the means ± SEMs. ns, not significant; *P < 0.05; **P < 0.01; ***P < 0.001; ****P < 0.0001. WT wild type, NS normal saline, AP acute pancreatitis, HPF high-powered field of view.

Macrophages are enriched in the pancreas in the early phase of AP, and their subtypes determine the severity [28–32]. It has also been reported that ischemic neurons with a background of *Mlkl* deletion could attenuate ischemic brain injury by promoting macrophage M2 polarization [6]. However, its role in AP remains unknown. We therefore constructed AP models in WT, *Mlkl*^-/-^ and *Ripk3*^-/-^ mice, and we found that a large number of macrophages infiltrated the pancreas of mice with AP (Fig. 4A, B, D, E). Interestingly, the knockout of *Mlkl* or *Ripk3* did not affect the total number of macrophages infiltrating the pancreas (Supplementary Fig. 5A, B). However, the polarization status of macrophages varied among mice with different genetic backgrounds. In mouse models of AP in WT and *Ripk3*^-/-^ mice, macrophages predominantly presented as the M1 subtype (F4/80+iNOS+ cells) accompanied by only a small number of M2 macrophages (F4/80+CD206+ cells) (Fig. 4D–F). Interestingly, macrophages were mainly polarized toward the M2 subtype in *Mlkl*^-/-^ mice with AP (Fig. 4A–C). Therefore, we speculated that the MLKL signaling pathway regulates the inflammatory response by mediating pancreatic macrophage polarization.

LPS can directly promote the polarization of macrophages into the M1 subtype [41]. In the present study, does LPS treatment lead to differences in results among WT, *Mlkl*^-/-^ and *Ripk3*^-/-^ mice with AP? First, mice were given 7 hourly intraperitoneal injections of saline plus one LPS at 10 mg/kg and were sacrificed 24 h after the last injection. The results showed that LPS alone was not able to induce inflammation (Supplementary Fig. 6A), macrophage enrichment or polarization in the pancreas (Supplementary Fig. 6B–F). Then, we constructed AP with cerulein alone (7 times, 50 μg/kg) in WT, *Mlkl*^-/-^ and *Ripk3*^-/-^ mice. The results showed that the pancreatic pathological scores (Supplementary Fig. 6A) and the abundance of macrophages (Supplementary Fig. 6B–F) of AP induced by cerulein alone were lower than those induced by cerulein plus LPS (Figs. 2A and 4A–C), and knockout of *Mlkl* or *Ripk3* did not attenuate AP (Supplementary Fig. 6A), consistent with what was observed by Boonchan [25]. Interestingly, the proportions of M1 and M2 subtypes in pancreatic macrophages were not significantly different among WT, *Mlkl*^-/-^ and *Ripk3*^-/-^ mice with AP induced by cerulein alone (Supplementary Fig. 6B–F). In addition, the proportions of M1 and M2 macrophages were close, unlike the predominance of M1 macrophages in the cerulein plus LPS-induced model (Fig. 4A–C). Therefore, we considered that LPS could significantly enhance the injury of the pancreas by cerulein, knockout of *Mlkl* was protective in this setting and affected the abundance and polarization status of infiltrating macrophages. However, LPS itself did not alter macrophage infiltration and polarization.

### *Mlkl* deletion in pancreatic acinar cells impairs their ability to promote macrophage M1 polarization

**Fig. 5:**
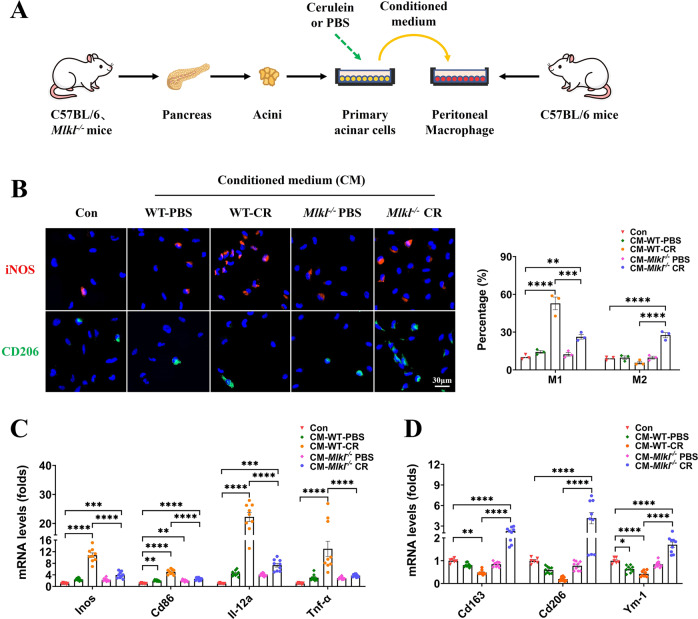
In the indirect coculture system of pancreatic acinar cells and peritoneal macrophages, conditioned medium from WT mouse-derived, cerulein-treated pancreatic acinar cells (CM-WT-CR) exhibited strong pro-M1 ability, while *Mlkl* deletion in pancreatic acinar cells impaired their ability to promote macrophage M1 polarization. **A** Schematic diagram for constructing an indirect coculture system of pancreatic acinar cells and peritoneal macrophages. **B** Immunofluorescence staining of iNOS (red) and CD206 (green) in peritoneal macrophages (*n* = 3). **C**, **D** mRNA levels of M1 macrophage markers (*Inos, Cd86, Il-12a, Tnf-α*) and M2 macrophage markers (*Cd163, Cd206, Ym-1*) in peritoneal macrophages. Data were normalized to GAPDH (*n* = 6-9). Values are shown as the means ± SEMs. ns, not significant; *P < 0.05; **P < 0.01; ***P < 0.001; ****P < 0.0001. WT wild type, Con control, CR cerulein, CM conditioned medium.

As we previously described, p-MLKL was predominantly localized to pancreatic acinar cells in the pancreas of mice with AP (Fig. 1D). To further explore the interaction between pancreatic acinar cells and macrophages in the setting of AP, we constructed an indirect coculture system (Fig. 5A). In immunofluorescence, iNOS and CD206 were used to mark M1 and M2 macrophages, respectively. In quantitative real-time PCR, the markers of M1 (*Inos, Cd86, Il-12a*, and *Tnf-α*) and M2 (*Cd163, Cd206*, and *Ym-1*) macrophages were consistent with previous studies [6, 30, 42–45]. Compared with the control group (Con), conditioned medium from WT and *Mlkl*^-/-^ mouse-derived, PBS-treated pancreatic acinar cells (CM-WT-PBS, CM-*Mlkl*^-/-^-PBS) exhibited weak pro-M1 ability, although significant differences were found only in CD86 and YM-1 mRNA levels (Fig. 5B–D). Conditioned medium from WT mouse-derived, cerulein-treated pancreatic acinar cells (CM-WT-CR) exhibited strong pro-M1 ability, and the proportion of iNOS+ cells reached 52.84 ± 3.68%, whereas CD206+ cells accounted for only 5.74 ± 1.93% (Fig. 5B). In addition, significant up- and downregulation was observed in the mRNA levels of M1 and M2 markers, respectively (Fig. 5C, D). Interestingly, conditioned medium from *Mlkl*^-/-^ mouse-derived, cerulein-treated pancreatic acinar cells (CM-*Mlkl*^-/-^-CR) exhibited moderate pro-M1 ability, with iNOS+ cells accounting for 26.30 ± 3.68%, while the proportion of CD206+ cells was elevated to 27.62 ± 1.93% (Fig. 5B). The mRNA levels of M1 markers in the CM-*Mlkl*^-/-^-CR group were significantly higher than those in the Con group but were significantly lower than those in the CM-WT-CR group, and correspondingly, a significant upregulation of M2 markers was observed (Fig. 5C, D).

### Differential gene expression was identified between WT and *Mlkl*^-/-^ mouse-derived, cerulein-treated pancreatic acinar cells

**Fig. 6:**
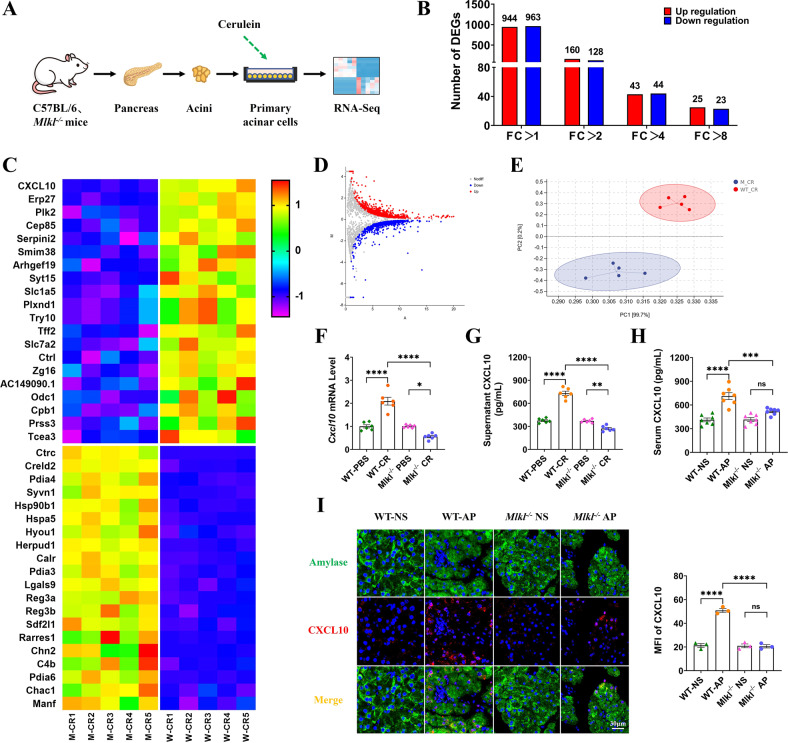
RNA sequencing was performed to identify the differentially expressed genes between WT and *Mlkl*^-/-^ mouse-derived, cerulein-treated pancreatic acinar cells. CXCL10 was the most significantly upregulated gene in pancreatic acinar cells from WT mice and was validated in cell and animal experiments. **A** Schematic diagram for preparing samples for RNA sequencing. **B** Number of differentially expressed genes (DEGs) at different fold changes. **C** Heatmap of the top 20 upregulated and downregulated genes. **D** Volcano plot of DEGs. **E** PCA plot. **F** mRNA levels of *Cxcl10* in pancreatic acinar cells isolated from WT and *Mlkl*^-/-^ mice. Data were normalized to GAPDH (*n* = 6). **G** Levels of CXCL10 in the supernatant of pancreatic acinar cells isolated from WT and *Mlkl*^-/-^ mice (*n* = 6). **H** Serum CXCL10 levels in WT and *Mlkl*^-/-^ mice (*n* = 7). **I** Immunofluorescence staining of amylase (green) colocalized with CXCL10 (red) in the pancreas of WT and *Mlkl*^-/-^ mice (*n* = 3). Values are shown as the means ± SEMs. ns, not significant; *P < 0.05; **P < 0.01; ***P < 0.001; ****P < 0.0001. WT wild type, NS normal saline, AP acute pancreatitis, CR cerulein, MFI mean fluorescence intensity.

Given that supernatants derived from WT and *Mlkl*^-/-^ mice-derived, cerulein-treated pancreatic acinar cells presented different properties in inducing macrophage polarization, we speculate that there are differentially expressed genes (DEGs) between them, which in turn lead to differences in the composition of their supernatants. Therefore, we isolated pancreatic acinar cells from WT and *Mlkl*^-/-^ mice and treated them with cerulein, followed by RNA sequencing (Fig. 6A). Many DEGs were identified (Fig. 6B–D), and the top 50 upregulated and downregulated genes are listed in Supplementary Tables 2 and 3, respectively. Principal component analysis (PCA) showed no overlap between the two groups, suggesting significant expression profile differences (Fig. 6E). The DEGs in the WT group with a fold change (FC) >1 included 944 upregulated and 963 downregulated genes. There were still 25 upregulated genes and 23 downregulated genes with an FC > 8 (Fig. 6B). Among many DEGs, interferon-γ-inducible protein 10 (CXCL10, also called IP-10) was the most significantly upregulated gene in the WT group (Log2FC = 3.57, FC = 11.87, *P* = 2.12E–59), which attracted our interest. CXCL10 belongs to the CXC chemokine family and is functionally categorized as an inflammatory chemokine that regulates immune responses [46]. CXCL10 can serve as a marker for M1-type macrophages [45, 47], and it has also been shown to induce M1 polarization of macrophages [48–50], but its role in AP remains poorly understood. We isolated pancreatic acinar cells to further validate the results of RNA sequencing. The cellular mRNA levels of *Cxcl10* (Fig. 6F) and CXCL10 in the cell supernatant (Fig. 6G) were significantly upregulated in WT mouse-derived, cerulein-treated pancreatic acinar cells, which is contrary to the result in pancreatic acinar cells with a background of *Mlkl* deletion. Similarly, serum CXCL10 (Fig. 6H) and pancreatic CXCL10 (Fig. 6I) were also significantly upregulated in WT mice with AP but remained unchanged in *Mlkl*^-/-^ mice with AP. The results indicated that the expression of CXCL10 was significantly affected by *Mlkl* deletion, and we hypothesized that this might account for the difference in its ability to induce macrophage polarization.

### Neutralization of CXCL10 in conditioned medium from WT mouse-derived, cerulein-treated pancreatic acinar cells impaired their pro-M1 ability

**Fig. 7:**
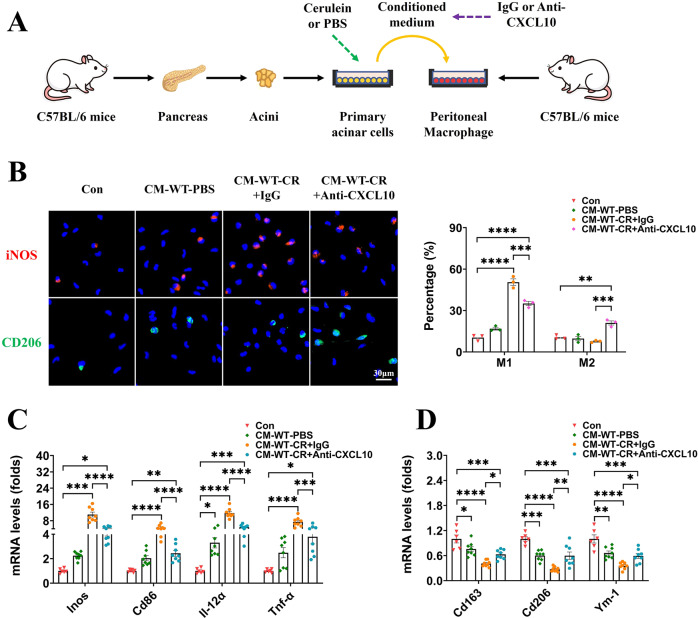
Rabbit anti-mouse CXCL10 neutralizing antibody (2 μg/mL) was added to the conditioned medium from WT mouse-derived, cerulein-treated pancreatic acinar cells. Rabbit IgG was added as an isotype control, and neutralization of CXCL10 impaired its ability to promote macrophage polarization toward M1. **A** Schematic diagram of constructing an indirect coculture system and preparing conditioned medium with CXCL10 neutralization. **B** Immunofluorescence staining of iNOS (red) and CD206 (green) in peritoneal macrophages (*n* = 3). **C**, **D** mRNA levels of M1 macrophage markers (*Inos, Cd86, Il-12a, Tnf-α*) and M2 macrophage markers (*Cd163, Cd206, Ym-1*) in peritoneal macrophages. Data were normalized to GAPDH (*n* = 6–8). Values are shown as the means ± SEMs. ns, not significant; *P < 0.05; **P < 0.01; ***P < 0.001; ****P < 0.0001. WT wild type, Con control, CR cerulein, CM conditioned medium.

CXCL10 was elevated in the supernatant of WT mouse-derived, cerulein-treated pancreatic acinar cells in our study, and it has been reported to promote macrophage polarization toward M1 [48–50]. Next, we explored whether neutralization of CXCL10 could alter its pro-M1 ability. Rabbit anti-mouse CXCL10 neutralizing antibody (2 μg/mL) was added to the conditioned medium from WT mouse-derived, cerulein-treated pancreatic acinar cells, and rabbit IgG was added as an isotype control (Fig. 7A). Consistent with our above findings, conditioned medium from WT mouse-derived, PBS-treated pancreatic acinar cells (CM-WT-PBS) exhibited weak pro-M1 properties (Fig. 7B, C). The isotype group (CM-WT-CR+IgG) exhibited strong pro-M1 properties, with the proportion of iNOS+ cells elevated to 50.59 ± 2.33% in immunofluorescence, as well as upregulation of M1 markers and downregulation of M2 markers in quantitative real-time PCR (Fig. 7B–D). Neutralization of CXCL10 (CM-WT-CR+Anti-CXCL10) impaired the ability to promote M1 polarization of macrophages, the proportion of iNOS+ cells (35.11 ± 2.33%) in immunofluorescence, and the expression of M1 markers in quantitative real-time PCR was still higher than in Con and CM-WT-PBS but already significantly lower than in CM-WT-CR+IgG. Correspondingly, there was an elevation in the proportion of CD206+ cells (21.01 ± 1.71%) and the expression of M2 markers in the CM-WT-CR+anti-CXCL10 group (Fig. 7B–D). Therefore, we concluded that the pro-M1 effect of conditioned medium from WT mouse-derived, cerulein-treated pancreatic acinar cells was partially achieved through the upregulation of CXCL10.

### In vivo neutralization of CXCL10 alleviated acute pancreatitis in mice by reducing macrophage M1 polarization

**Fig. 8:**
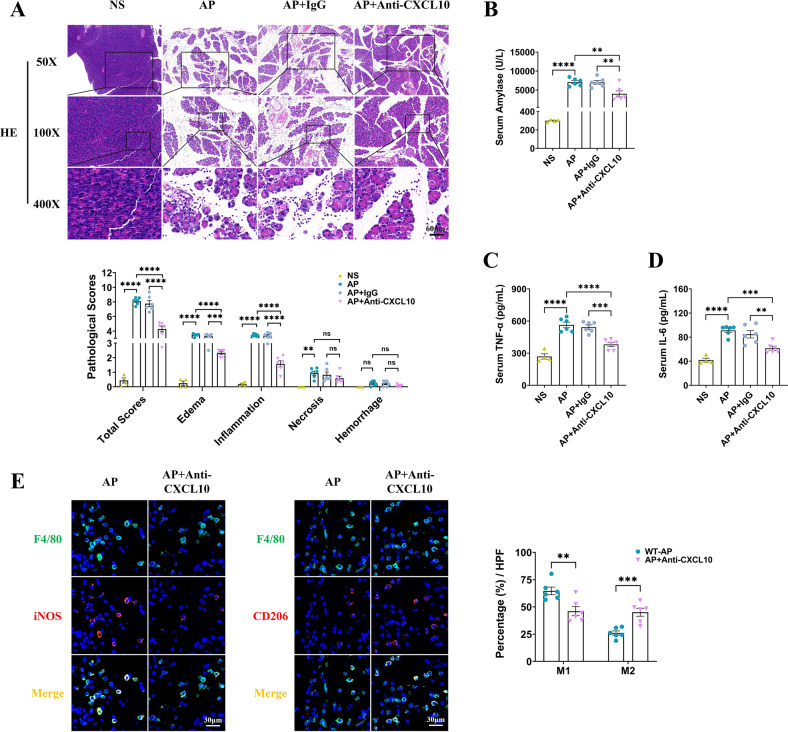
Rat anti-mouse CXCL10 dissolved in saline was administered after the first cerulein injection in C57BL/6J mice (20 μg/each), while rat IgG was injected as an isotype control. In vivo neutralization of CXCL10 alleviated AP in mice by reducing macrophage M1 polarization. **A** HE staining and pancreatic pathological scores of the pancreas (*n* = 6). **B** Serum amylase (*n* = 6). **C**, **D** Serum TNF-α and IL-6 (*n* = 6). **E** Immunofluorescence staining of F4/80 (green) colocalized with iNOS (red) or CD206 (red) in the pancreas of mice and the percentage of macrophages (*n* = 6). Values are shown as the means ± SEMs. ns, not significant; *P < 0.05; **P < 0.01; ***P < 0.001; ****P < 0.0001. NS normal saline, AP acute pancreatitis.

Given that we have shown in vitro that CXCL10 in the supernatant of pancreatic acinar cells promoted macrophage polarization toward M1, we were curious whether in vivo neutralization of CXCL10 could attenuate AP in mice. 20 μg of rat anti-mouse CXCL10 dissolved in saline was intraperitoneally administered after the first cerulein injection in C57BL/6J mice, while rat IgG was injected as an isotype control. The results showed that pancreatic edema and inflammatory cell infiltration in HE staining (Fig. 8A) as well as serum amylase (Fig. 8B) levels were all alleviated after the administration of a neutralizing antibody against CXCL10. The serum inflammatory mediators TNF-α and IL-6 in mice with AP were significantly elevated and remained unchanged after IgG administration, whereas neutralization of CXCL10 significantly downregulated the serum levels of TNF-α and IL-6 (Fig. 8C, D), suggesting a diminished systemic inflammatory response. Furthermore, administration of anti-CXCL10 reduced the number of M1 macrophages and increased the number of M2 macrophages in the pancreas of mice with AP (Fig. 8E). Thus, in vivo neutralization of CXCL10 attenuated the severity of AP by limiting pancreatic local inflammation and the systemic inflammatory response.

## Discussion

AP is a local-to-systemic variable inflammatory response caused by various stimuli, including excessive alcohol consumption and gallstone disease. Although the prognosis of patients with AP has improved, the current treatment remains unspecific and supportive due to a lack of understanding of the complex cellular pathways and immune events that occur during AP. Macrophages are the most abundant immune cells infiltrating the pancreas and contribute to the inflammatory responses in AP. A deeper understanding of the interplay between injured acinar cells and infiltrating macrophages would assist in exploring novel therapeutic targets.

In the present study, we found that MLKL and its phosphorylated protein p-MLKL were upregulated in the pancreas of a cerulein-induced mouse AP model and cerulein-treated pancreatic acinar cells, independent of its canonical upstream molecule *Ripk3*. CaMKII may be a novel upstream molecule of *Mlkl* in this setting. Knockout of *Mlkl* attenuated AP in mice by reducing the polarization of pancreatic macrophages toward the M1 phenotype, and this protective effect was partly achieved by reducing the secretion of CXCL10 from pancreatic acinar cells, whereas knockout of *Ripk3* did not. In vitro neutralization of CXCL10 impaired the pro-M1 ability of conditioned medium from WT mouse-derived, cerulein-treated pancreatic acinar cells, whereas in vivo neutralization of CXCL10 reduced the polarization of pancreatic macrophages toward M1 and the severity of AP in mice.

The RIPK3-MLKL-necroptosis axis is the canonical necroptotic signaling pathway that contributes to disease pathogenesis in acute kidney injury [4], ischemic stroke [6], ischemia‒reperfusion injury of steatotic livers [51], psoriasis [52], colitis [53], etc. However, many noncanonical mechanisms of RIPK3 and MLKL have been reported in recent years. For example, RIPK3 can induce necroptosis in dasatinib-induced cardiotoxicity independently of *Mlkl* but dependent on intracellular *Hmgb1* [54]. MLKL can also trigger necroptosis independently of *Ripk3* in tanshinol A-induced lung cancer cell death [55], anti-CD147 antibody-induced hepatocellular carcinoma cell death [16], radiation-induced antitumor immunity [56], and autoimmune hepatitis [57]. It is worth mentioning that RIPK3 or MLKL may also mediate pathways independent of cell death. Inhibition of RIPK3 attenuated cardiac ischemia‒reperfusion injury [58] and sepsis [59] by reducing mitochondrial dysfunction, and inhibition of the RIPK1-RIPK3 pathway reduced tumor cell metastasis by decreasing vascular endothelial permeability [60]. Furthermore, MLKL attenuated colon inflammation and colitis tumorigenesis via suppression of inflammatory responses [17], inhibited intestinal tumorigenesis by suppressing the IL-6/JAK2/STAT3 pathway [61] and promoted cellular differentiation in myeloid leukemia by facilitating the release of G-CSF [18]. Inhibition of MLKL prevented obesity-induced insulin resistance in mice [20] and promoted intracellular Listeria replication [62]. Therefore, in the context of inflammatory and neoplastic diseases, RIPK3 and MLKL do not necessarily represent the onset of necroptosis, and their roles will need to be assessed in each disease model individually. In our study, we found that MLKL is involved in the pathogenesis of AP by modulating pancreatic macrophage polarization and appears to be independent of *Ripk3* and cell death.

The role of MLKL and RIPK3 in AP varies between studies. Boonchan reported that the knockout of *Mlkl* was not protective against experimental murine AP and that the knockout of *Ripk3* instead worsened disease severity [25]. In contrast, Wu et al. found that *Mlkl* deficiency protected mice from cerulein-induced AP [26]. In addition, Louhimo’s results showed that the knockout of *Ripk3* could alleviate AP induced by taurocholate or cerulein in mice [21]. He et al. [23] and Zhang et al. [24] observed that knockout of *Ripk3* alleviated cerulein-induced AP in mice. There is also a study suggesting that the knockout of *Ripk3* did not affect the severity of caerulein-induced AP in mice [22]. The role of Nec-1, a RIPK1 inhibitor, in AP is also controversial [21, 63, 64]. Most studies [21–26] used cerulein to induce AP in mice, so we checked whether LPS treatment in the present study caused the difference in results, since LPS can activate TLR4 and its downstream targets NF-κB and MAPK [38, 39] and can directly activate caspase11 [40]. However, the results suggested that LPS did not cause a difference in the severity of AP in mice through the above pathways in the present study. Therefore, we considered that the methods in which AP in mice was constructed, the period of observation, and the dose and frequency of cerulein administration varied among these studies, perhaps explaining the variability in results. Another possible reason for the difference in results may be the usage of mice (sex, age and littermate usage). In the present study, mice were sex-matched, 8–10 weeks of age and littermate controlled, whereas Boonchan et al. [25] used female mice, Wu et al. [26], He et al. [23] and Newton et al. [22] used male mice, and Louhimo et al. [21] and Zhang et al. [24] used a nonsex-selected design. Boonchan et al. [25], Louhimo et al. [21], and Newton et al. [22] did not use littermates, whereas Wu et al. [26], He et al. [23] and Zhang et al. [24] used littermates for the experiments. The mice also ranged in age from 6 to 12 weeks. Moreover, as discussed above, RIPK3 and MLKL are involved in various pathological and physiological processes, including but not limited to cell death. Therefore, these results are all valuable because they may represent AP with different etiologies, courses, and severities, which provide evidence for further elucidation of the pathogenesis of AP, particularly the role of MLKL.

We performed RNA-seq and experimental validation to find that the knockout of *Mlkl* reduced CXCL10 release from cerulein-stimulated pancreatic acinar cells and impaired their ability to induce macrophage polarization toward M1, which may be one of the mechanisms underlying its protective effect against AP. Several studies support our findings. Zhang et al. confirmed that CXCL10 can induce macrophage polarization toward the M1 phenotype [65]. Tsai et al. reported that pulmonary fibroblast-secreted CXCL10 polarized alveolar macrophages toward the M1 phenotype and mediated inflammatory responses [49]. Zhang et al. found that MyD88 activation in HSCs increased the secretion of CXCL10, which promoted macrophage M1 polarization and aggravated liver fibrosis [66]. Moreover, some studies have used CXCL10 as a marker to identify M1 macrophages [45, 47, 67, 68]. Thus, targeting pancreatic acinar cell-released CXCL10 may be a promising anti-inflammatory therapy against AP.

In summary, the present findings suggest that *Mlkl*-dependent release of CXCL10 from pancreatic acinar cells and subsequent macrophage polarization to M1 are critical for the progression of AP in mice. Targeting the MLKL-CXCL10-macrophage axis might be a novel strategy for the treatment of AP. Certainly, if the same findings were to be observed in patients with AP, it would greatly enhance its clinical translational value.

## Materials and methods

### Antibodies and reagents

Cerulein (HY-A0190) was purchased from MedChemExpress (Shanghai, China). The neutralizing antibody against CXCL10 (GTX31179) and rabbit IgG isotype control (GTX35035) for in vitro experiments were purchased from GeneTex (Shanghai, China), while the neutralizing antibody against CXCL10 (MAB466) and rat IgG isotype control (6-001-F) for in vivo experiments were purchased from R&D Systems (Shanghai, China).

Primary antibodies against IL-6 (GB11117), TNF-α (GB11188), and F4/80 (GB11027) were purchased from Servicebio (Wuhan, China). RIPK3 (DF7339, used in western blot), p-MLKL (AF3902, used in immunofluorescence and immunohistochemical staining), p-CaMKII (AF3493), p-p38 MAPK (AF4001), and p-NF-κB p65 (AF2006) were purchased from Affinity (Liyang, China). Cleaved Caspase11 p20 (sc-374615) was purchased from Santa Cruz (Shanghai, China). p-MLKL (ab196436, used in western blot) and p-RIPK3 (ab222320, used in western blot) were purchased from Abcam (Shanghai, China). Amylase (bs-4030R) and CXCL10 (bs-1502R) were purchased from Bioss (Beijing, China). iNOS (13120) and p-RIPK3 (91702, used in immunofluorescence staining) were purchased from Cell Signaling Technology (Shanghai, China). CD206 (60143-1-Ig), GAPDH (10494-1-Ig), and MLKL (66675-1-Ig, used in western blot) were purchased from Proteintech (Wuhan, China). The specificity of the p-MLKL signal must be carefully addressed, since Samson has observed a band in *MLKL*^*-/-*^ mouse cells using p-MLKL (ab196436) [69]. Therefore, we validated p-MLKL (AF3902) and p-MLKL (ab196436) using *Mlkl*^-/-^ tissues. The results showed that p-MLKL (AF3902) and p-MLKL (ab196436) exhibited satisfactory specificity in immunohistochemical staining and western blot, respectively (Supplementary Fig. 7). The specificity of MLKL (66675-1-Ig) has been validated by Miyata in the tissue of *Mlkl*^*-/-*^ mice [70].

Secondary antibodies: HRP-goat anti-rabbit IgG (H+L) (GB23303), Cy3-conjugated goat anti-rabbit IgG (H+L) (GB21303), Cy3-conjugated donkey anti-mouse IgG (H+L) (GB21401), FITC-conjugated donkey anti-rabbit IgG (H+L) (GB22403), and FITC-conjugated goat anti-mouse IgG (H+L) (GB22301) were purchased from Servicebio (Wuhan, China).

### Mice

C57BL/6J (WT) mice were purchased from Hunan SJA Laboratory Animal Co., Ltd. The *Ripk3*^-/-^ and *Mlkl*^-/-^ mice were generous gifts from Dr Ben Lu. All experimental mice were sex-matched and 8–10 weeks of age. Mice were housed under specific pathogen-free (SPF) conditions with a 12-h alternating day and night environment and fed standard laboratory chow and water ad libitum. All animal experiments were approved by the Institutional Animal Care and Use Committees of Central South University.

### Acute pancreatitis (AP) model

Mouse models of AP were induced by injection of cerulein (CR) plus lipopolysaccharide (LPS) as previously described [32, 33]. In brief, WT, *Ripk3*^-/-^, and *Mlkl*^-/-^ littermates were randomly allocated without blinding and fasted overnight. Mice in the experimental groups (*n* = 6) were given 7 hourly intraperitoneal injections of CR at 50 μg/kg as well as one intraperitoneal injection of LPS at 10 mg/kg immediately after the last injection of CR. Mice in the control groups (*n* = 4–6) received the same volume of saline. All mice were sacrificed 24 h after the last injection. Blood and pancreatic tissue were collected for subsequent experiments.

### Isolation of mouse pancreatic acinar cells and preparation of conditioned medium

Pancreatic acinar cells were isolated from WT, *Ripk3*^-/-^, and *Mlkl*^-/-^ mice by methods previously described [71, 72]. Briefly, the freshly collected pancreas was digested by collagenase P (11249002001, Roche, Germany) in the presence of 0.1 mg/mL soybean trypsin inhibitor (T6414, Sigma, USA). Then, the acini were filtered through a 100-μm cell strainer and purified by sedimentation through 4% bovine serum albumin (BSA) in Dulbecco’s modified Eagle’s medium (DMEM). Finally, cells were resuspended in DMEM containing 0.1 mg/mL soybean trypsin inhibitor and 1% BSA and seeded in 6-well plates. Acinar cells were treated with cerulein (100 nM) for 1 h to induce acinar cell injury, and the control group was treated with an equal volume of PBS. The cells were then collected by centrifugation for RNA or protein extraction, and the supernatant was collected for ELISA or used as the conditioned medium (CM) for peritoneal macrophages.

### Mouse peritoneal macrophage isolation and intervention

Mouse peritoneal macrophages were obtained according to a previous study [73]. In brief, female C57BL/6J mice were intraperitoneally injected with 3 mL of 3% thioglycolate. Three days later, the mice were sacrificed and intraperitoneally injected with 10 mL of PBS to harvest peritoneal exudate cells and then centrifuged at 1200 rpm for 5 min. The cells were resuspended, incubated in 6-well plates and cultured with 2 mL of DMEM containing 10% fetal bovine serum and 1% penicillin and streptomycin. Nonadherent cells were removed 2 h later, and the remaining cells were used as peritoneal macrophages.

To explore the effect of pancreatic acinar cells on peritoneal macrophages, we constructed an indirect coculture system. In brief, 400 μL of CM prepared from pancreatic acinar cells was added and incubated for 24 h, and the cells were then harvested for western blot, quantitative real-time PCR, and immunofluorescence staining.

### Pancreas weight/body weight (PW/BW) ratio

The body weight and pancreas weight of mice were recorded when harvested. The results were expressed as milligrams of pancreas per gram of body weight as previously described [74].

### Measurement of amylase, TNF-α, IL-6, and CXCL10 in supernatant/serum

Blood samples were stored at 4 °C overnight and centrifuged at 1200 × g for 10 min, and then the serum was collected. Primary pancreatic acinar cells were collected at the indicated time points and centrifuged at 1200 rpm/min for 5 min, and the supernatant was collected for subsequent assays.

Serum amylase levels were quantified using a fully automatic biochemistry analyzer with standard techniques (Chemray 240, Redu Life Technologies, Shenzhen, China). TNF-α (CSB-E04741 m) and IL-6 (CSB-E04639 m) levels in serum and CXCL10 (CSB-E08183 m) levels in serum and cell supernatant were measured using commercial enzyme-linked immunosorbent assay (ELISA) kits (CUSABIO, Wuhan, China).

### Histological analysis

Mice were euthanized by CO_2_ inhalation, and then the pancreas was rapidly removed and fixed in 4% paraformaldehyde for 24 h. The paraffin-embedded tissues were sectioned (5 μm) and used for hematoxylin and eosin staining (HE). Ten random images of each pancreatic HE slice were selected and scored individually by two professional pathologists who were blinded to the experimental protocol. The histology score was evaluated based on Schmidt criteria [75], which include edema, necrosis, inflammation, and hemorrhage. The final score was expressed as the average of these values.

### Immunofluorescence staining

Paraffin sections (5 μm) were deparaffinized using xylene and absolute ethanol and hydrated using ethanol (95%→85%→75%), followed by antigen retrieval with Tris-EDTA buffer (G1201, Servicebio, Wuhan, China). QuickBlock™ Blocking Buffer was applied for blocking (P0260, Beyotime, Shanghai, China), and then a spontaneous fluorescence quenching agent (G1221, Servicebio, Wuhan, China) was added. The sections were incubated with primary antibodies at 4 °C overnight and secondary antibodies for 1 h at room temperature. A Tyramide signal amplification kit (G1235-100T, Servicebio, Wuhan, China) was used for fluorescent double-label staining. Finally, nuclei were stained using DAPI. The sections were observed using a fluorescence microscope. The number of F4/80+iNOS+ and F4/80+CD206+ cells as well as the mean fluorescence intensity (MFI) of p-RIPK3, p-MLKL, or CXCL10 were calculated by ImageJ 1.52.

For immunofluorescence staining of primary peritoneal macrophages, we prepared cell-attached slides. Briefly, the slides were placed into 6-well plates in advance, and peritoneal lavage fluid was added. The nonadherent cells were removed after 2 h, and the remaining cells adherent to the slides were peritoneal macrophages, which could be used for subsequent staining after interventions. The staining procedure was performed as described above.

### Immunohistochemical staining

Paraffin sections (5 μm) were used for immunohistochemical staining. The procedures for deparaffinization, hydration, antigen retrieval, and blocking were the same as those described for immunofluorescence staining. Hydrogen peroxide (3%) was applied to block endogenous peroxidase activity. The sections were incubated with primary antibodies at 4 °C overnight and secondary antibodies for 1 h at room temperature. Then, the DAB working solution (ZLI-9017, ZSGB-BIO, Beijing, China) was added dropwise, and the positive area was brownish yellow under the microscope. Finally, hematoxylin was used to counterstain the nuclei. The mean optical densities (IODs) of IL-6 and TNF-α were measured by Image-Pro Plus 6.0.

### RNA extraction and quantitative real-time PCR

Total RNA of primary pancreatic acinar cells and peritoneal macrophages was extracted using a Quick RNA Extraction Kit (AG21023, Accurate Biotech, Changsha, China) according to the manufacturer’s instructions. RNA purity and concentration were analyzed using a Nanodrop 2000 (Thermo Fisher Scientific). cDNA was synthesized using HiScript II Q RT SuperMix for qPCR (R223-01, Vazyme, Nanjing, China). Next, qRT‒PCR was performed using ChamQ Universal SYBR qPCR Master Mix (Q711-02, Vazyme, Nanjing, China). The primers are listed in Supplementary Table 1.

### Protein extraction and western blot

Pancreatic tissues or primary pancreatic acinar cells were homogenized and lysed in RIPA buffer (BD0031, Bioworld, Nanjing, China) containing protease inhibitor (CW2200S, CWbiotech, Taizhou, China) and phosphatase inhibitor (CW2383S, CWbiotech, Taizhou, China), and debris was removed by centrifugation at 14,000 × g for 10 min at 4 °C. The denatured proteins were electrophoresed in SDS‒PAGE gels followed by transfer onto PVDF membranes (Millipore, USA). The membranes were blocked with 5% skimmed milk for 1 h and further incubated with primary antibody solution at 4 °C overnight and secondary antibody solution for 1 h at room temperature. Finally, the immunostained bands were detected via a hypersensitive chemiluminescence solution (P1052, Applygen, Beijing, China). The relative expression of proteins of interest was calculated by ImageJ 1.52.

### RNA-seq and analyses

Total RNA was extracted from primary pancreatic acinar cells using TRIzol Reagent (R401-01, Vazyme, Nanjing, China), after which the concentration, quality, and integrity were determined using a NanoDrop spectrophotometer (Thermo Fisher Scientific). Three micrograms of RNA was used as input material for the RNA sample preparations. Sequencing libraries were generated using the TruSeq RNA Sample Preparation Kit (Illumina, San Diego, CA, USA). The sequencing library was then sequenced on a HiSeq platform (Illumina) by Shanghai Personal Biotechnology Cp. Ltd.

### Neutralization of CXCL10 in vitro and in vivo

For the neutralization of CXCL10 in the conditioned medium from pancreatic acinar cells, rabbit anti-mouse CXCL10 neutralizing antibody (2 μg/mL) was added, while rabbit IgG was added as an isotype control. For in vivo neutralization of CXCL10, 20 μg of rat anti-mouse CXCL10 dissolved in saline was intraperitoneally administered after the first cerulein injection, while rat IgG was injected as an isotype control.

### Statistical analysis

All data are expressed as the means ± SEMs. Two-tailed Student’s *t* test was performed for comparisons between two groups, and one-way ANOVA followed by Tukey’s test was used for multiple comparisons. Statistical analysis was performed with GraphPad Prism 8.1 software. A *p* value less than 0.05 was considered statistically significant.

## Supplementary information

Supplementary Figure Legends

Supplementary Figure 1

Supplementary Figure 2

Supplementary Figure 3

Supplementary Figure 4

Supplementary Figure 5

Supplementary Figure 6

Supplementary Figure 7

Supplementary Table Legends

Supplementary Table 1

Supplementary Table 2

Supplementary Table 3

aj-checklist

Original images of western blot

## Data Availability

The datasets used and/or analyzed during the current study are available from the corresponding author upon reasonable request.
