# Supplementary material for: MLKL signaling regulates macrophage polarization in acute pancreatitis through CXCL10
Source: Cell Death Dis. 2023 Feb 24;14(2):155. doi: 10.1038/s41419-023-05655-w (PMC9958014; doi:10.1038/s41419-023-05655-w)
Supplement: Supplementary file 1 — Supplementary Figure Legends [file 41419_2023_5655_MOESM1_ESM.docx]

**Supplementary Figure Legends:**

**Supplementary Figure 1** (**A**) MFI of p-MLKL and p-RIPK3 in immunofluorescence staining (n=3). (**B**) Mean IOD of p-MLKL in immunohistochemical staining (n=3). (**C-D**) The ratio of p-MLKL/GAPDH and p-MLKL/MLKL, as well as p-RIPK3/GAPDH and p-RIPK3/RIPK3 in Western blot of mouse pancreas (n=6). (**E-F**) The ratio of p-MLKL/GAPDH and p-MLKL/MLKL, as well as p-RIPK3/GAPDH and p-RIPK3/RIPK3 in Western blot of pancreatic acinar cells (n=7). (**G**) Mean IOD of p-MLKL in immunohistochemical staining (n=6). (**H**) The ratio of p-MLKL/GAPDH and p-MLKL/MLKL in western blot of mouse pancreas (n=6). (**I**) The ratios of p-MLKL/GAPDH and p-MLKL/MLKL in western blot of pancreatic acinar cells (n=7). Values are shown as the means ± SEMs. ns, not significant; *P< 0.05; **P< 0.01; ***P< 0.001; ****P< 0.0001. MFI, mean fluorescence intensity; IOD, integrated optical density; WT, wild type; NS, normal saline; AP, acute pancreatitis; CR, cerulein.

**Supplementary Figure 2** The expression of p-CaMKII was upregulated in the pancreas of mice with AP and colocalized with p-MLKL. (**A**) Immunohistochemical staining of p-CaMKII in the pancreas of mice (n=3-4). (**B**) Immunofluorescence staining of p-MLKL (green) colocalized with p-CaMKII (red) in the pancreas of mice (n=3-4). Values are shown as the means ± SEMs. ns, not significant; *P< 0.05; **P< 0.01; ***P< 0.001; ****P< 0.0001. IOD, integrated optical density; NS, normal saline; AP, acute pancreatitis.

**Supplementary Figure 3** (**A-B**) Serum levels of IL-6 and TNF-α were measured by ELISA in WT, *Mlkl^-/-^*, *Ripk3^-/-^* mice with AP and their controls (n=4). Values are shown as the means ± SEMs. ns, not significant; *P< 0.05; **P< 0.01; ***P< 0.001; ****P< 0.0001. WT, wild type; NS, normal saline; AP, acute pancreatitis.

**Supplementary Figure 4** The expression of p-p65, p-p38 and cleaved caspase-11 was upregulated in the pancreas of mice with AP, and knockout of *Mlkl* or *Ripk3* did not reduce the activation of these pathways. (**A**) Immunohistochemical staining of p-p65 and p-p38 in the pancreas of mice (n=3-4). (**B**) Immunohistochemical staining of cleaved caspase-11 in the pancreas of mice (n=3-4). Values are shown as the means ± SEMs. ns, not significant; *P< 0.05; **P< 0.01; ***P< 0.001; ****P< 0.0001. IOD, integrated optical density; WT, wild type; NS, normal saline; AP, acute pancreatitis.

**Supplementary Figure 5** (**A**) Count of total macrophages in WT and *Mlkl^-/-^* mice with AP and their controls (n=6). (**B**) Count of total macrophages in WT and *Ripk3^-/-^* mice with AP and their controls (n=6). Values are shown as the means ± SEMs. ns, not significant; *P< 0.05; **P< 0.01; ***P< 0.001; ****P< 0.0001. WT, wild type; NS, normal saline; AP, acute pancreatitis; HPF, high-powered field of view.

**Supplementary Figure 6** (**A**) HE staining and pancreatic pathological scores of the pancreas (n=3-4). (**B-C**) Immunofluorescence staining of F4/80 (green) colocalized with iNOS (red) or CD206 (red) in the pancreas (n=3-4). (**D**) Count of total macrophages. (**E-F**) Percentage and cell counts of M1 and M2 macrophages. Values are shown as the means ± SEMs. ns, not significant; *P< 0.05; **P< 0.01; ***P< 0.001; ****P< 0.0001. WT, wild type; NS, normal saline; AP, acute pancreatitis; HPF, high-powered field of view.

**Supplementary Figure 7** (**A**) Immunohistochemical staining of p-MLKL in the pancreas of *Mlkl^-/-^* mice. (**B**) Western blot of p-MLKL in the pancreas, liver and lung of WT and *Mlkl^-/-^* mice. GAPDH was used as the loading control. WT, wild type; NS, normal saline; AP, acute pancreatitis.
