# Supplementary figures and images for: MLKL signaling regulates macrophage polarization in acute pancreatitis through CXCL10
Source: Cell Death Dis. 2023 Feb 24;14(2):155. doi: 10.1038/s41419-023-05655-w (PMC9958014; doi:10.1038/s41419-023-05655-w)

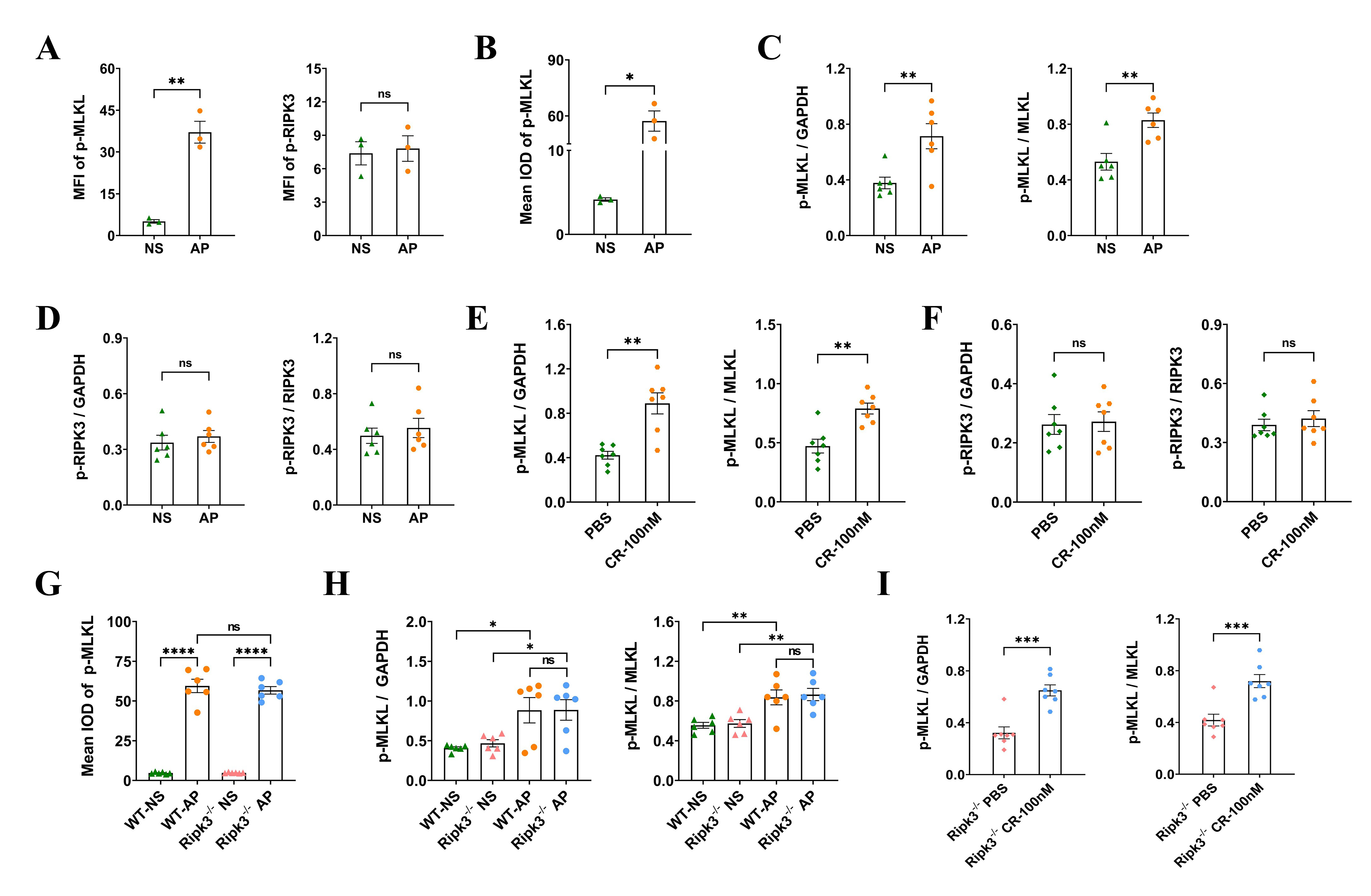

Supplement: Supplementary file 2 — Supplementary Figure 1 [file 41419_2023_5655_MOESM2_ESM.jpg]

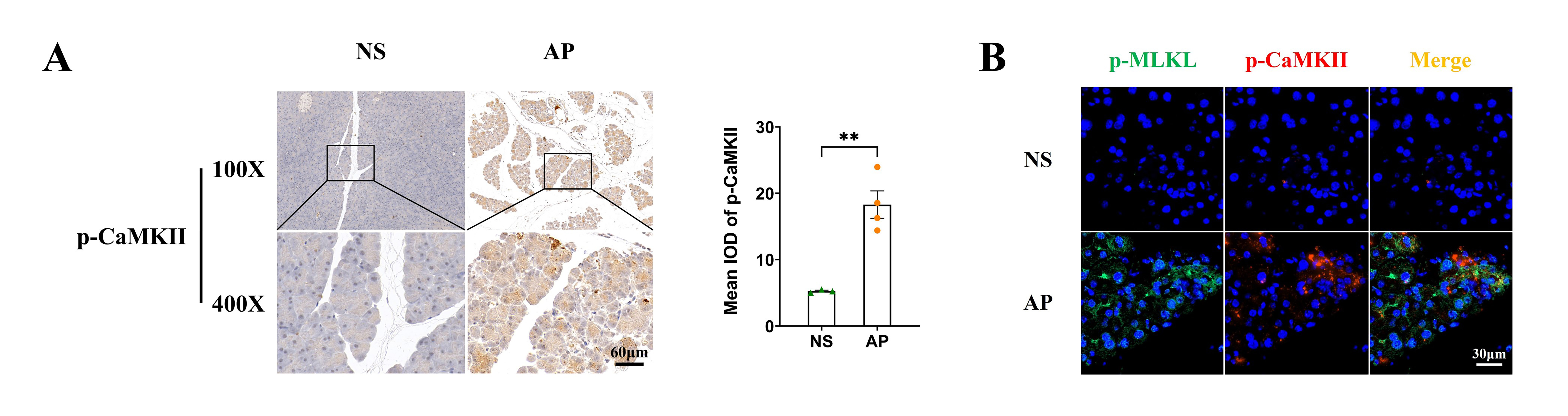

Supplement: Supplementary file 3 — Supplementary Figure 2 [file 41419_2023_5655_MOESM3_ESM.jpg]

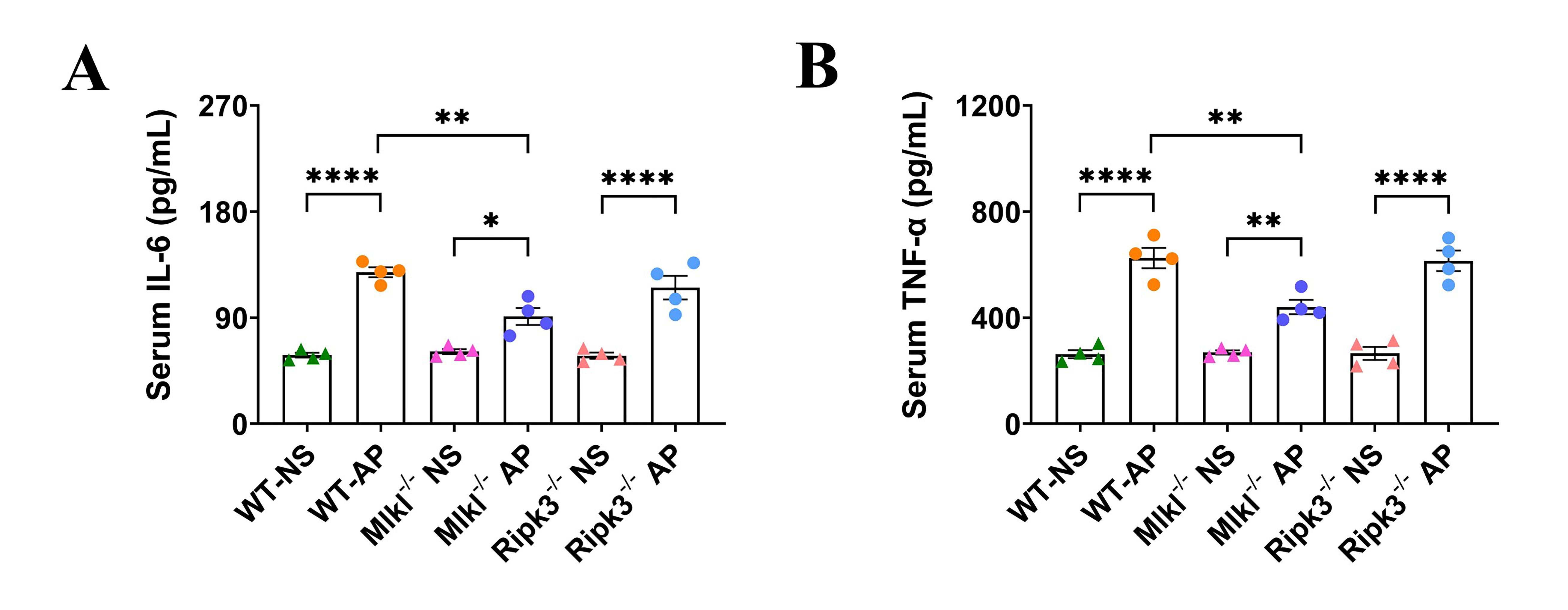

Supplement: Supplementary file 4 — Supplementary Figure 3 [file 41419_2023_5655_MOESM4_ESM.jpg]

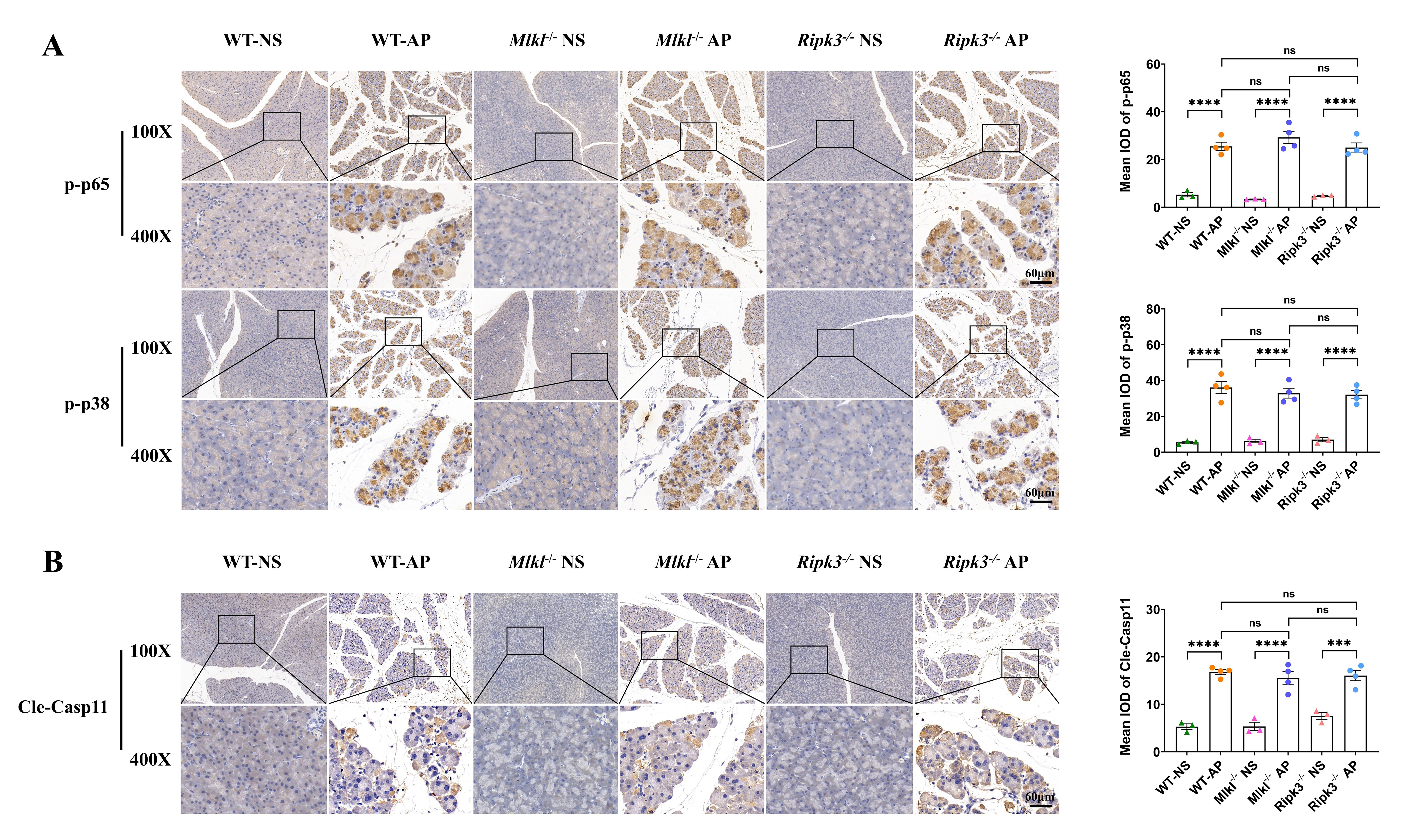

Supplement: Supplementary file 5 — Supplementary Figure 4 [file 41419_2023_5655_MOESM5_ESM.jpg]

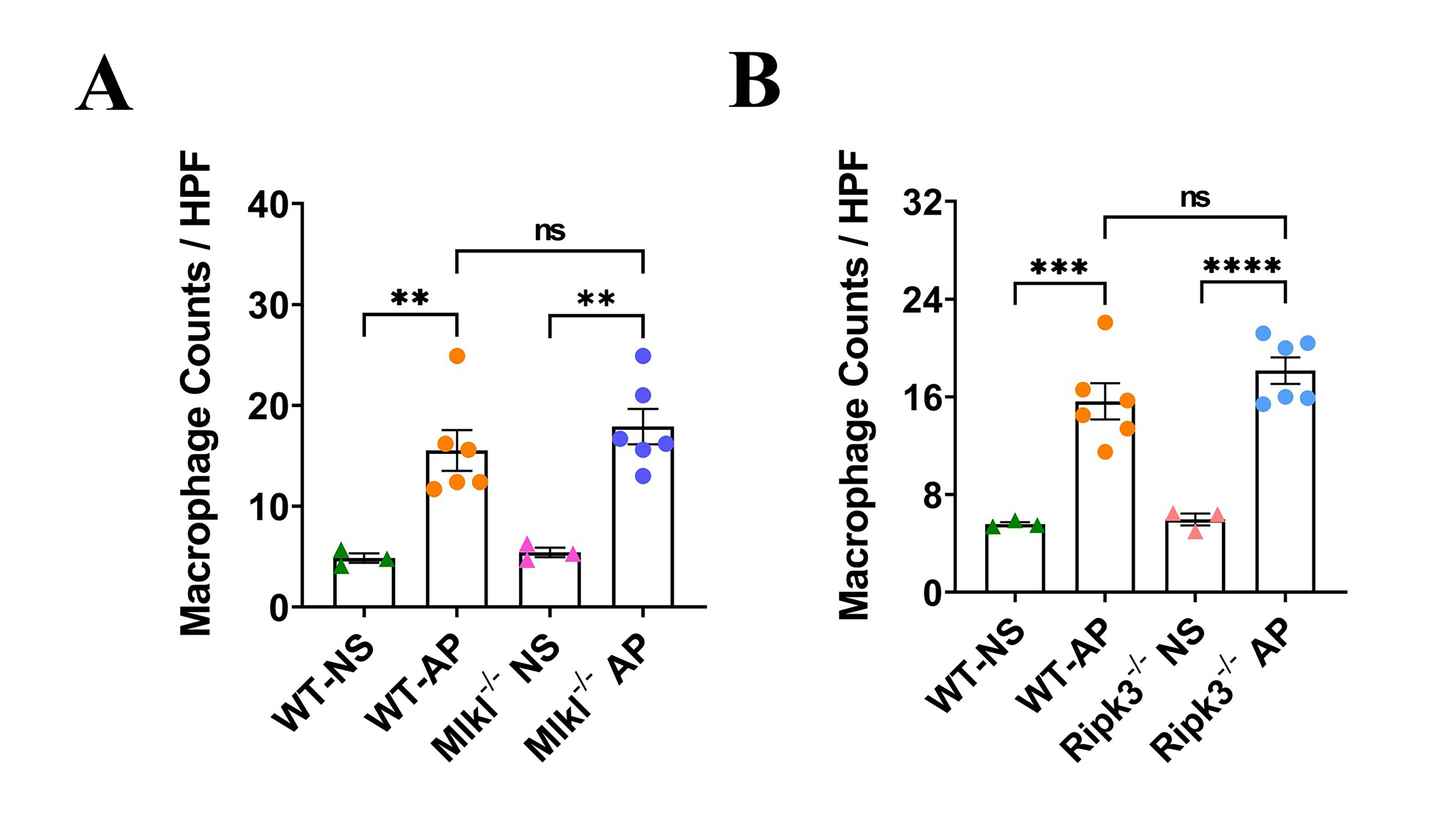

Supplement: Supplementary file 6 — Supplementary Figure 5 [file 41419_2023_5655_MOESM6_ESM.jpg]

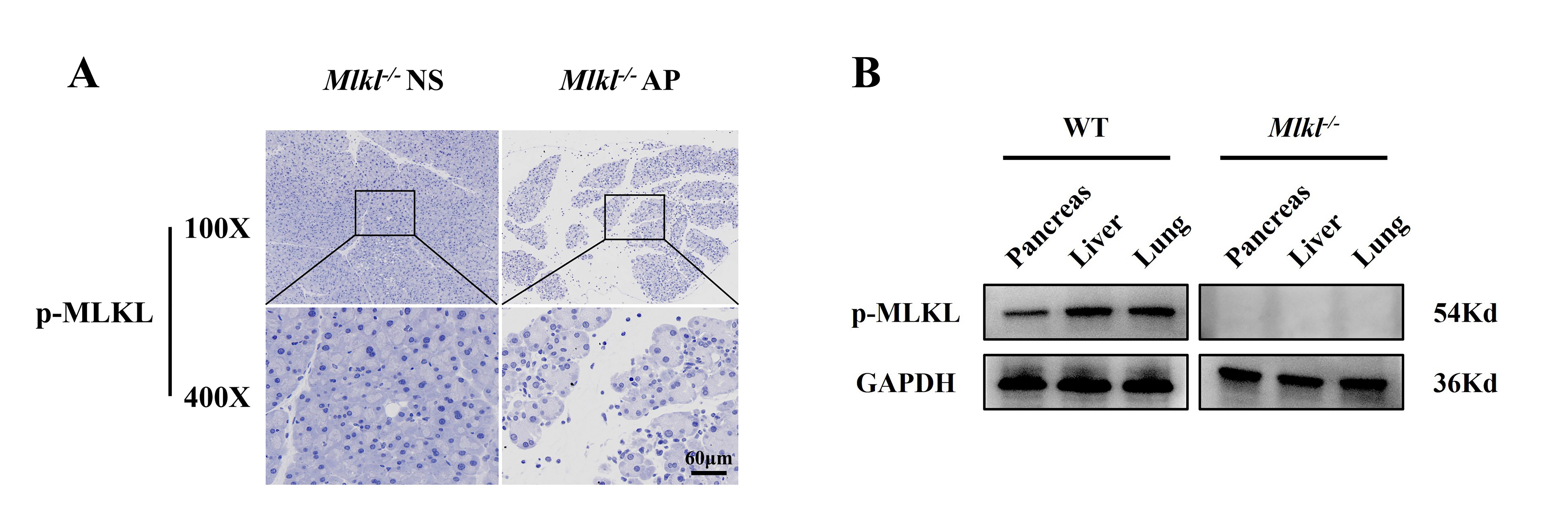

Supplement: Supplementary file 8 — Supplementary Figure 7 [file 41419_2023_5655_MOESM8_ESM.jpg]
