# Supplementary material for: MLKL signaling regulates macrophage polarization in acute pancreatitis through CXCL10
Source: Cell Death Dis. 2023 Feb 24;14(2):155. doi: 10.1038/s41419-023-05655-w (PMC9958014; doi:10.1038/s41419-023-05655-w)
Supplement: Supplementary file 9 — Supplementary Table Legends [file 41419_2023_5655_MOESM9_ESM.docx]

**Supplementary Table Legends:**

**Supplementary Table 1** Sequences of the primers used for quantitative real-time PCR.

**Supplementary Table 2** The top 50 upregulated differentially expressed genes.

**Supplementary Table 3** The top 50 downregulated differentially expressed genes.
