# Supplementary material for: MLKL signaling regulates macrophage polarization in acute pancreatitis through CXCL10
Source: Cell Death Dis. 2023 Feb 24;14(2):155. doi: 10.1038/s41419-023-05655-w (PMC9958014; doi:10.1038/s41419-023-05655-w)
Supplement: Supplementary file 10 — Supplementary Table 1 [file 41419_2023_5655_MOESM10_ESM.docx]

**Table 1 Sequences of the primers used for quantitative real-time PCR**

| **Gene** | **Forward primer (5′–3′)** | **Reverse primer (5′–3′)** |
| --- | --- | --- |
| TNF-α | ACCCTCACACTCACAAACCA | AGCCTTGTCCCTTGAAGAGA |
| iNOS | GCAGAGTGAAAAGTCCAGCC | TCCACAACTCGCTCCAAGAT |
| CD206 | CTGCAAGGAAGGTTGGCATT | CCCAGTCCAGGCATTGAAAG |
| GAPDH | TGGTCCTCAGTGTAGCCCAAG | CTGCCCAGAACATCATCCCT |
| CD86 | CGGATGGTGTGTGGCATATG | AGCAGCATCACAAGGAGGAG |
| Ym1 | TCTGAATGAAGGAGCCACTGA | CCACGGCACCTCCTAAATTG |
| RIPK3 | GCACAGGACACATCAGTTGG | CGTGTCTTCCATCTCCCTGA |
| MLKL | CACAGAATTCCATCAGCCGG | CTTTCCAGTGGCAATTTCCCA |
| IL-12a | ACTTCTTCCACAACAAGAGGGAG | CCAGCATGCCCTTGTCTAGAAT |
| CD163 | GGAGCTGGTTCTGGACCAAT | CAGATCTGCTCCCTCTAAGCA |
| CXCL10 | ACGTGTTGAGATCATTGCCAC | GTCGCACCTCCACATAGCTT |
